# Empirical evidence for the functional benefit of intra-specific wing shape variation in a sedentary bird, the Oriental Magpie (*Pica serica*)

**DOI:** 10.1038/s41598-025-13894-4

**Published:** 2025-08-12

**Authors:** Seokbong Chae, Jusun Hwang, Jae Chun Choe, Piotr G. Jablonski, Sang-im Lee, Jooha Kim

**Affiliations:** 1 Department of Mechanical Engineering, Johns Hopkins University, Baltimore, MD USA; 2 National Institute of Wildlife Disease Control and Prevention, Gwangju, Korea; 3 Laboratory of Behavior and Ecology, Division of Ecoscience, Ewha Womans University, Seoul, Korea; 4 Laboratory of Behavioral Ecology and Evolution, School of Biological Sciences, Seoul National University, Seoul, Korea; 5 Museum and Institute of Zoology, Polish Academy of Sciences, Warsaw, Poland; 6 Department of New Biology, Daegu Gyeongbuk Institute of Science and Technology, Daegu, Korea; 7 Department of Mechanical Engineering, Ulsan National Institute of Science and Technology, Ulsan, Korea

**Keywords:** Behavioural ecology, Evolutionary ecology

## Abstract

This study investigates the intraspecific variation in wingtip shape and its effects on aerodynamic forces and flight capabilities with the Oriental Magpies as a model species. Characterized by short and rounded wings, Oriental Magpies are highly sedentary and exhibit wingtip shape variations between juveniles and adults, as well as between males and females due to physiological changes during breeding. Analysis of 115 individuals revealed a significant interaction between sex and age in the location of the wingtip, with adult females exhibiting wings with backward-shifted wingtips than other sex and age categories. In order to examine the functional aspect of this pattern of variation, we conducted wind tunnel experiments and measured the aerodynamic performances of three wings by varying the position of wingtip from forward to backward. The results show that wings with backward-shifted wingtips have higher lift coefficient compared to wings with forward-shifted wingtips, especially at low free-stream velocities. Our findings suggest that wings with backward-shifted wingtips enhance maneuverability during both turning- and straight-flight conditions, particularly during slow gliding flight. We hypothesize that aerodynamic benefits of the backward-shifted wingtips are more important for adult females, who has increased body weight with center of mass shifted to rear part of the body due to fully developed reproductive organs including eggs and follicles. Our results suggest that age- and sex-dependent wingtip shape change can be fine-tuned according to intraspecific variation in the ecological requirements of the individuals.

## Introduction

The wing shape of birds has been regarded as an adaptation for their flight efficiency. Interspecific variation in the wing shape and its association with the ecological niche of the species has well been documented. In general, long-distance migratory species typically exhibit a high aspect ratio and low wing loading (= body mass/wing area), which may reduce the energetic costs during the migration^1^. In contrast, non-migratory and sedentary species tend to have a relatively lower aspect ratio, which is interpreted as a morphological adaptation to their specific foraging strategies^1^. Wing morphology is also closely linked to the type of maneuverability required during flight^2^. For instance, high-speed fixed-wing maneuvering is essential for pure coursers such as swifts, and these species typically possess pointed wings. In contrast, low-speed flapping maneuvering is prominent in pure hawkers and low-flying birds in dense habitat, which often exhibit more rounded wings^3^.

Wingtip morphology, particularly the chordwise position of the longest primary feather relative to the leading edge, has also been reported to be closely associated with the ecological demands of birds^4^. In this study, we refer to wings where the longest primary feather is positioned closer to the leading edge as having a forward-shifted wingtip, and those where it is positioned further toward the trailing edge as having a backward-shifted wingtip. Migratory birds generally exhibit forward-shifted wingtips compared to non-migratory species^4^. In contrast, backward-shifted wingtips are more frequently observed in non-migratory species, a pattern that has been interpreted as an adaptation for enhanced takeoff performance, which may, in turn, reduce predation risk^4,5^.

Similar logic can be applied to intraspecific variation in the wingtip morphology when ecological difference is present among the individuals within a species. So far, intraspecific variation in the wing shape has been associated with differences in ecological requirements of the individuals, and studies have reported variation related to sex and/or age of the individuals^6–8^, the dispersal or migration distance of the individuals or populations (citations below), and environmental factors such as vegetation or elevation^9–11^. Non-migratory populations within a given species tend to possess relatively backward-shifted wingtips, a trait that generates greater lift in flapping and facilitates rapid takeoff, which are essential to escape from predators^12,13^. In contrast, in many passerine species, individuals that undertake long-distance dispersal, such as juveniles or migratory populations, tend to possess forward-shifted wingtips^14–16^.

With the exception of a few observations (e.g.,^3,17^), these findings broadly support a convergent hypothesis that forward-shifted wingtips are better suited for fast and sustained flight, whereas backward-shifted wingtips are more effective for generating high lift in flapping and enabling rapid takeoff. This relationship suggests that the wingtip position from the leading edge is strongly coupled to the ecological demands and flight performance requirements of the species. However, the aerodynamic effects of such wingtip position remain insufficiently explored and have yet to be directly validated through quantitative measurements of aerodynamic forces (i.e., drag and lift).

Among factors contributing to intraspecific variation in wing shape, the interaction between sex and age remains less well studied, particularly in non-migratory sedentary birds. Considering that males and females go through different physiological changes due to breeding, the interaction between sex and age on the wing shape can be expected. In adult females, who invest substantial amount of resources to the egg production, the center of mass is shifted backward when they carry developed follicles and eggs. Notably, the flight performance of adult females significantly deteriorates during the breeding season due to an increase in the weight of ovaries and eggs and a decrease in pectoral muscle mass^18–20^. These imply that the functional aspect of wing shape variation is likely to depend on the variation in the ecological requirements of individuals.

**Fig. 1:**
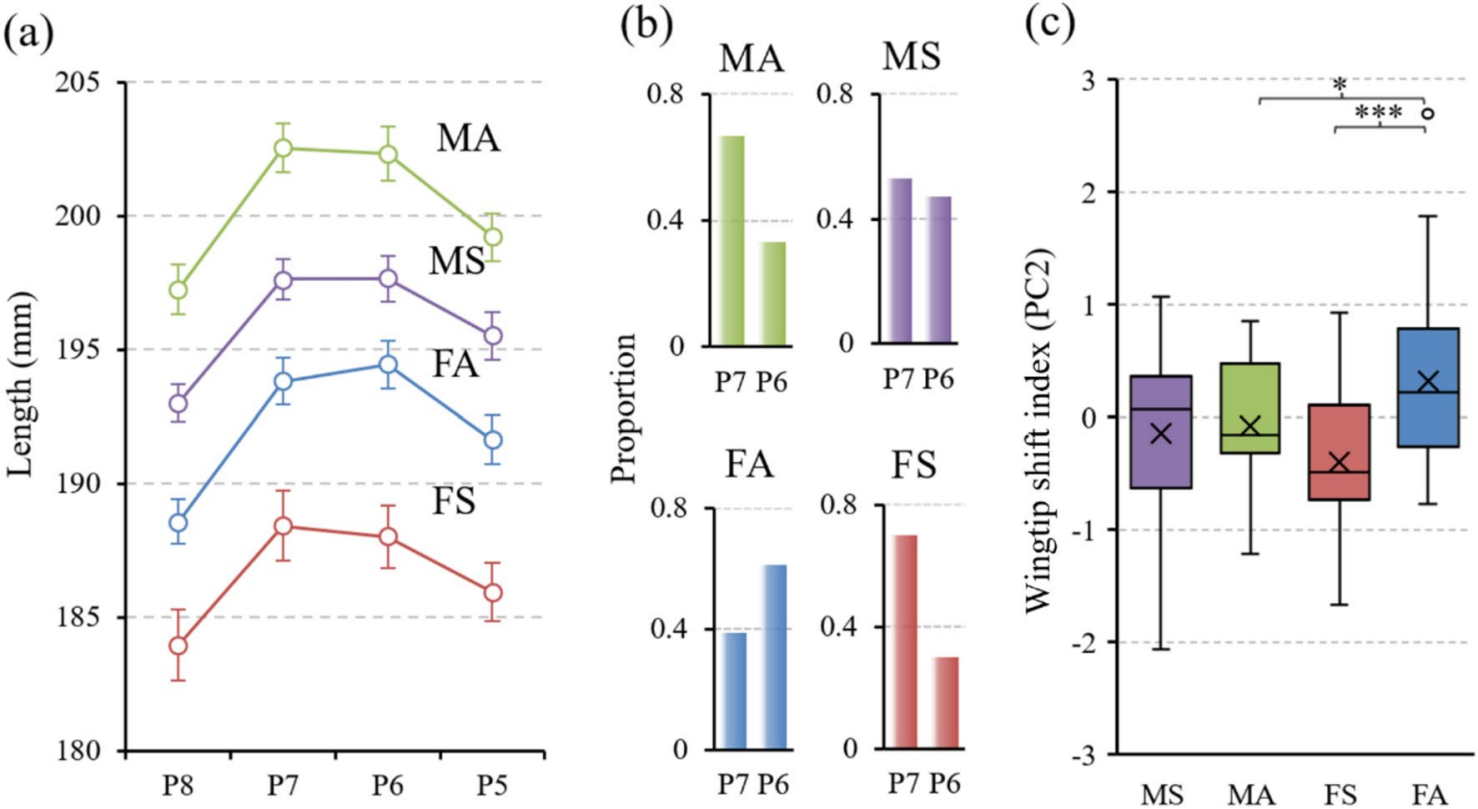
Wingtip shape variation in the Oriental magpies (n = 115). The lengths of the primary feathers forming the wingtip (8th–5th primaries) vary depending on the sex and the age of individuals (**a**). Only adult females show different wingtip shape which is also visible in the proportion of individuals having the wingtips on the 6th (P6) versus 7th (P7) primary feathers (**b**). Wingtip shift index, the second principal component (PC2) extracted from the primary distances, shows that the females exhibit significant wing shape change with age while males do not (**c**). MS, MA, FS and FA denote second-year male, after-second-year male, second-year female and after-second-year female respectively. Statistical significance of the Tukey–Kramer post-hoc test is given as the asterisks; ‘*’ indicates 0.01 < *P* < 0.05 and ‘***’ indicates *P* < 0.001.

In this study, we aimed to reveal the functional aspect of the intraspecific variation in the wingtip shape of the Oriental Magpie (*Pica serica*). Magpies (genus *Pica*) generally have short and rounded wings, with longest primary (wingtip) located at the 7th or 6th primary (corresponding to 3rd or 4th feather from the leading edge). The average distance of natal dispersal, which is known to be slightly female-biased, is larger than that of movement of the breeding adults (425 m vs 25 m^21^). Moreover, juveniles form non-breeding flocks in the winter and the home range of such non-breeding flocks can be ten times as large as territory size of adults (30–50 ha and 5 ha respectively, measured in European species^21^). Thus, following the speculation that wings with a forward-shifted wingtip are better suited for longer migration, we predicted that juveniles would exhibit wings with forward-shifted wingtips than adults. Also, as in many other bird species, breeding females carry well-developed follicles and developing eggs in the body prior to egg-laying. Based on the increased load in the caudal part of the body, we predicted that adult females would benefit from having wings with backward-shifted wingtips, which would shift the aerodynamic center backward (see Supplementary Fig. 1) for enhancing flight stability^19,22^. Examination of wing shape of 115 individuals of the Oriental Magpie (60 adults and 55 juveniles; 62 males and 53 females) reveal that indeed show significant age difference in wing shape in the females only (Fig. 1a); compared to males and young females, adult females had more rounded wings with backward-shifted wingtip (larger PC2 values, GLMM, sex*age, F_1,107_ = 5.83, *P* = 0.0175; for the details with the wing shape characterization, refer to Supplementary Table 1 and associated figures), with the wingtip more frequently located at the 6th primary feather, thus positioned farther from the leading edge (Fig. 1b,c).

**Fig. 2:**
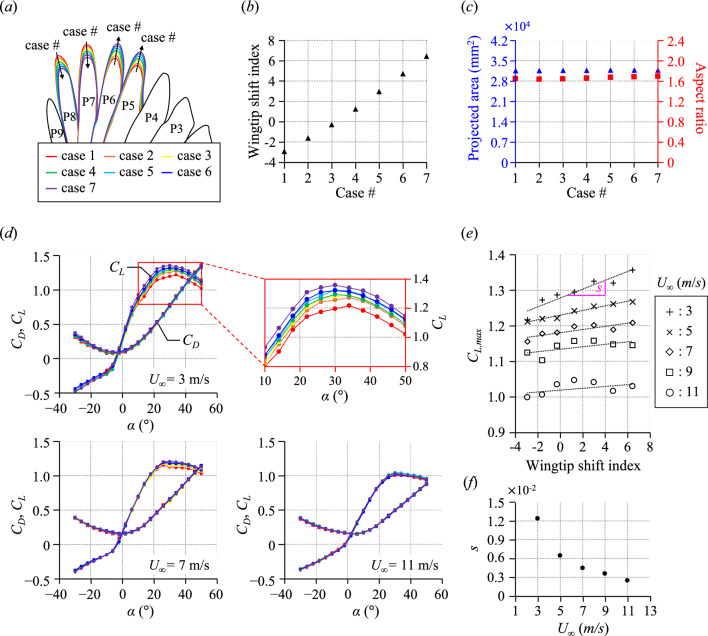
Effects of wingtip shape on wingtip shift index, projected area, aspect ratio, and aerodynamic force coefficients for wing 1. (**a**) Variations in the wingtip shape depending on the case number; (**b**,**c**) Variations in (**b**) wingtip shift index (black triangle) and (**c**) projected area (blue triangle) and aspect ratio (red square) depending on the case number; (**d**) Variations in the drag and lift coefficients with the angle of attack for various case numbers and free-stream velocities (*U*_∞_ = 3 m/s, 7 m/s, and 11 m/s); (**e**) Variations in the maximum lift coefficient with the wingtip shift index for the free-stream velocities from 3 to 11 m/s. Here, the dash-line in (**e**) represent the linear trend line for each free-stream velocity; (**f**) A variation in the slope (*s*) of the linear trend line in (**e**) with the free-stream velocity.

In order to test the functional benefit of wings with a backward-shifted wingtip, we conducted wind tunnel experiments on dried wing models (wing 1, wing 2, and wing 3) equipped with a device adjusting the lengths of distal primary feathers (see the details in Method and Supplementary Fig. 2). Seven wingtip shapes (from case 1 to case 7) were implemented by adjusting the lengths of the 5th to 8th primary feathers (P5 to P8), which significantly affect the wingtip shift index (see Fig. 2a and Supplementary Fig. 3). The wingtip shift index quantifies the chordwise position of the wingtip, with larger values indicating a backward-shifted wingtip. Note that the details of the feather length changes are described in Supplementary Fig. 3(b). For all three wing models, as the case number increases, the wingtip shift index increases linearly, indicating wingtip shifted backwards, without any siginifcant changes in the projected area and aspect ratio (Fig. 2b,c for wing 1; Supplementary Fig. 3c–e for other models). The variations in the wingtip shift index for the wing models are within the range observed in actual magpie wings. Thus, the difference in any aerodynamic performance observed in the wing models used in this study can be mostly attributed to the manipulated wingtip shift index. Figure 2d shows the variations of drag coefficient (*C*_*D*_ = *D*_*W*_/0.5*ρU*_∞_^2^*A*_*W*_; *D*_*W*_ = wing drag, *ρ* = air density, *U*_∞_ = free-stream velocity, *A*_*W*_ = projected area of the wing) and lift coefficient (*C*_*L*_ = *L*_*W*_/0.5*ρU*_∞_^2^*A*_*W*_; *L*_*W*_ = wing lift) with the angles of attack (*α*) for wing 1 at various *U*_∞_s. At the lowest *U*_∞_ of 3 m/s, as the case number increases (i.e., the wingtip shift index increases), the lift coefficient increases pronouncedly in the high-angle-of-attack region of approximately 10° or more. This means that the wingtip shift index directly affects the lift coefficient of the wing, with a backward-shifted wingtip (i.e., a higher wingtip shift index) providing a higher lift coefficient in the high-angle-of-attack region. For the drag coefficient, however, no noticeable difference was observed depending on the case number in the overall range of angle of attack. As *U*_∞_ increases, the increase in lift coefficient with the case number gradually reduces, with little change in lift coefficient with the case number at the highest* U*_∞_ of 11 m/s. To quantitatively understand the effect of the wingtip shape on the aerodynamic performance, we examined the variation in the maximum lift coefficients with the wingtip shift index (see Fig. 2e). The maximum lift coefficient increased by ~ 12% (from 1.22 to 1.36) at *U*_∞_ = 3 m/s as the case number increases from 1 to 7, having a high slope (*s*) of the linear trend line of 1.2 $\times$ 10^–2^ as shown in Fig. 2f. As *U*_∞_ increases to 11 m/s, the value of *s* substantially decreased by about 80%, increasing the maximum lift coefficient within 2% with the wingtip shift index. These aerodynamic effects of the wingtip shape were observed similarly in the other wings (see Supplementary Figs. 5−7). Our results show that the change in wingtip shape has a significant effect only on the lift coefficient at the low free-stream velocity region, especially at the high angle-of-attack region. As *U*_∞_ gradually increases, the primary feathers can oscillate quickly, bend, and fail to maintain their shape, so that aerodynamic effects by the wingtip shape are no longer sustained.

**Fig. 3:**
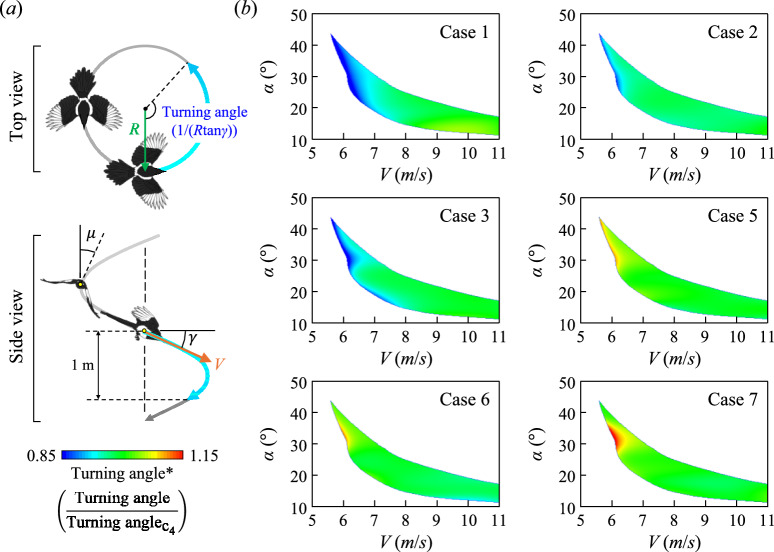
Effects of the wingtip shape on the turning-flight performance (turning angle). (**a**) Description of the flight trajectory under the helical descent (this figure was created based on Fig. 1a in Lentink et al.^23^, with modifications.) The turning angle is defined as the angular displacement of the flight path during a 1 m vertical descent. (**b**) Change in normalized turning angle depending on glide speeds and angles of attack for various case numbers in the turning-flight environment. Here, the turning angles are normalized to those of the natural wingtip shape (i.e. case 4).

Being highly sedentary, magpies’ flight frequently involves short turning glide. Also, magpies usually forage on the ground which inevitably involves many descending flights with substantial maneuverability. To evaluate the flight performance of the wing models in turning- and straight-flight conditions, the flight trajectory was simulated for the magpie model with wing 1. We estimated glide angle (*γ*), bank angle (*μ*), and turn radius (*R*) using the lift, drag, and weight of the wing- and body-dried model under the assumption of helical-descending flight with a constant gliding speed, following Lentink et al.^23^ (details are described in Fig. 3a and Method). Note that details of drag measurements for the body model are in Supplementary Fig. 8.

During turning, three turning indices, including turning angle (1/(*R*tan*γ*)), angular velocity (*V*cos*γ*/*R*), and curvature (1/*R*), were evaluated and presented in Fig. 3b and Supplementary Fig. 9. Here, the turning angle is defined as the angular displacement of the flight path during a 1 m vertical descent, the angular velocity represents the rate of change of the turning angle over time, and the curvature corresponds to the curvature of the circular flight path. Figure 3b presents the contours of the normalized turning angle (turning angle*) at various angles of attack and glide speeds (*V*) depending on the wingtip shape. It should be noted that turning angle* is defined as the turning angle normalized by that of the natural wingtip shape (i.e., case 4) under the same angle of attack and glide speed. To maintain stable free-descent conditions, the glide speed has a minimum threshold of 5.6 m/s, and the range of the angle of attack gradually decreases as the glide speed increases. Although direct comparison is not possible, the range of flight speed and attack angles is roughly congruent with Tobalske and Dial^24^ observation on the flapping flight of the magpie in the wind tunnel, where average body angle was reported as ~ 27 degrees in 4 m/s and ~ 17 degrees in 6 m/s. Deviations in turning angles from those of the natural wingtip shape are visible only in the low-glide-speed region (7 m/s or less approximately),forward-shifted wingtips (i.e., cases 1–3) result in turning angles smaller than the natural wingtip shape (i.e., case 4), whereas backward-shifted wingtips (i.e., cases 5–7) allow greater turning angles. For instance, at *V* = 6.2 m/s and *α* = 30$^\circ$, the turning angle for case 7 (i.e., the wing with the most backward-shifted wingtip) is greater by approximately 27% compared to that for case 1 (i.e., the wing with the most forward-shifted wingtip). Turning velocity and the curvature also show similar tendencies, with backward-shifted wingtips bringing enhanced turning performances only in a low glide speed range (Supplementary Figs. 9). These results show that the overall turning-flight performance enhances as the wingtip shifts backward.

**Fig. 4:**
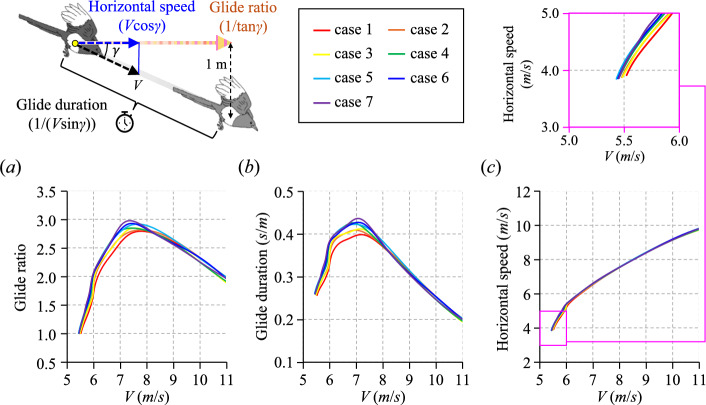
Effects of the wingtip shape on the straight-flight performance. (**a**–**c**) Variations in (**a**) glide ratio, (**b**) glide duration, and (**c**) horizontal speed with the glide speed for wing 1 with various case numbers. Here, the inset on the up-right side shows the enlarged view of the pink square in (**c**). The glide ratio denotes the horizontal distance traveled per 1 m of vertical descent; the glide duration refers to the time required to descend 1 m vertically; and the horizontal velocity represents the forward speed component projected onto the horizontal plane.

Assuming a straight flight condition with an infinite turn radius (*R* ~ ∞), the glide ratio (1/tan*γ*), glide duration (1/(*V*sin*γ*)), and horizontal velocity (*V*cos*γ*) were calculated and are presented in Fig. 4. Here, the glide ratio represents the horizontal flight distance per 1 m vertical descent; the glide duration is defined as the time required to descend 1 m vertically; and the horizontal velocity corresponds to the forward speed component projected along the horizontal plane. As shown in Fig. 4a,b, both the glide ratio and glide duration increase with *V*, reaching a peak at *U*_∞_ = 7 m/s approximately, and then they gradually decrease with increasing *V*. In the *V* range less than 8 m/s approximately, glide ratio and duration have a trend of being higher with increasing the case number (i.e., as the wingtip shifted backwards). Therefore, the peak values of the glide ratio and duration increase by about 8% and 10% as the case number increases from 1 to 7. When *V* increases more than about 8 m/s, on the other hand, there are no significant changes in the glide ratio and duration according to the case number. For the horizontal velocity, as shown in Fig. 4c, it gradually increases with* V*. Differences in the horizontal velocity with the wingtip shift index are prominent only at a very low *V* range. For instance, at *V* = 5.52 m/s, the horizontal velocity increases by about 7% as the case number increases from 1 to 7. However, this difference in the horizontal velocity becomes less prominent with increasing *V*, becoming less than 1% after about 7 m/s. These results indicate that all straight-flight performance indices exhibit higher values as the wingtip shift index increases only in the low-glide-speed regime, which is the similar result with those in the turning flight described in Fig. 3. Based on the aerodynamic and flight performance estimations derived from wind tunnel experiments, wings with a high wingtip shift index exhibit a higher lift coefficient and demonstrate superior flight performance under both turning- and straight-flight conditions. These findings partially align with hypotheses proposed in previous studies on inter- and intra-specific variations in wing shape, particularly those suggesting that wings with backward-shifted wingtips provide advantages in maneuverability and lift generation. However, this study did not find evidence supporting the notion that wings with forward-shifted wingtips confer advantages for sustained long-distance flights. This discrepancy may stem from the assumption of gliding flight throughout the analysis. Given that adult magpies frequently engage in short, intermittent gliding flights within their territories, our findings suggest that the wing morphology with backward-shifted wingtips in adult females enhances flight efficiency in such scenarios. Conversely, longer-distance movements, such as natal dispersal or the movements of non-breeding flocks typically performed by younger individuals, likely involve extended periods of flapping flight, a mode not considered in this study.

If the flight mode differs mostly by the age of the birds, then one would find a difference in wingtip shape between young and old individuals. However, male magpies did not show significant age-related change in the wingtip shape. This indicates the importance of breeding-related physiological change in the female. During the breeding season, increased weight of the eggs and reproductive organs deteriorates the flight performance^19^. Freshly laid magpie egg weighs on average 10–12 g per egg, which corresponds to 5–6% of the average body mass of the females. We currently do not have data for testes mass, and we estimate its average weight to be approximately 5 g (based on Blanco and de la Puente^25^ volume, assuming the density of testes tissue is 1 g/mm^3^), which corresponds to ~ 2% of the average body mass of adult males. Apart from the difference in loading, increased mass locates more into the dorsal part for the males (on top of the kidney) and more into the dorso-ventral part for the females (as the egg develops it moves down toward the cloaca, and the fully grown egg stays in the uterus for 20 h which is located closer to the cloaca; according to Proctor and Lynch^26^). Thus, we think that the increased mass due to developed reproductive organs and their location would be substantially different between adult females and males. Although the females may not be very active before and during egg laying, well developed reproductive organs, including enlarged ovary and follicles, uterus, and shell glands would altogether increase substantial mass even before the egg is developed inside the body. Thus, the females would experience increased mass in the posterior part of the abdomen well before the egg laying, during which they extensively forage outside the nest and are exposed to predation risk. Moreover, the pectoral muscle mass of adult females decreases during the egg formation^18,27^. These deterioration in the flight performance can be interpreted as a decreased ability to escape from predators^28^. We suggest that greater modification in wingtip shape of females after the first complete molt than males is linked with their impaired flight ability during the breeding season, and having backward-shifted wingtips may compensate for the impaired flight. For instance, at a flight speed of 7 m/s, the most backward-shifted wingtip configuration (i.e., case 7) of wing 1 produces additional lift equivalent to approximately 10 g of body weight compared to the most forward-shifted configuration (i.e., case 1). Given that birds can experience a body mass increase of approximately 7% during the breeding season^19^, which corresponds to around 15 g in magpies, this additional lift appears sufficient to partially compensate for the flight performance deterioration caused by the increased body mass.

Another possibility, not mutually exclusive to above hypothesis, is that the wingtip shape may serve as a mechanism for the balance between stability and agility. According to previous research, it is known that for many species, the aerodynamic center (i.e., neutral point in^22^) of the wings is located behind the center of gravity, and this arrangement is inherently stabilized for gliding flight^22,29^. In contrast, agility, which includes the ability to perform linear accelerations (axial agility) and angular accelerations (torsional agility), is inversely related to stability because greater moments are required to overcome stabilizing moments^30^. Since the position of the wingtip can directly influence the aerodynamic center (refer to Supplementary Fig. 1), the morphology of the wingtip may have evolved to provide an appropriate balance between stability and agility depending on ecological demands. In female adults, the increase in the weight of the egg and reproductive organs causes the center of mass to shift backward, which can lead to a decrease in static stability. Our findings suggest that a corresponding shift in wingtip position toward the rear may move the aerodynamic center backward as well, helping to maintain the distance between the aerodynamic center and the center of mass (i.e., static margin) and thereby compensating for the potential loss of stability.

In birds, not only the wings but also the tail generate both lift and drag, contributing to flight performance and stability^29,31,32^. Magpies have long graduated tails, which are known to primarily generate drag for flight stability and can directly influence the aerodynamic center^29^. Changes in lift and drag caused by variations in wingtip shape, as well as shifts in the aerodynamic center, may also affect tail angle and shape during flight. For example, individuals with forward-shifted wingtips may require increased drag from the tail to maintain stability, which could potentially reduce forward velocity. Understanding how tail morphology and its dynamic adjustment during flight impact overall performance deserves further investigation, especially across different ages and sexes.

In interspecific variation, it has been observed that birds having long-distance migrations tend to have forward-shifted wingtips^33–36^. In contrast, non-migratory species tend to have backward-shifted wingtips, which provide fast takeoff, essential for escaping predators^12^. Based on our results, it is predicted that the flight performance under both straight and turning flights is enhanced as the wingtip becomes backward-shifted, and that adult females, having impaired flight capabilities, would be benefited by having backward-shifted wingtips. Whether similar patterns would be observed in other species warrants more study. Even though the breeding-related physiological change would be commonly experienced by the females in any avian species, how much the wingtip shape change can benefit the females going through such changes may depend on the life history and the wing shape of the species. For instance, it may not be feasible for migratory species, which generally possess wings with a high aspect ratio and a forward-shifted wingtip, to achieve age-related changes in wingtip morphology, or migration may exert much stronger selective pressures on the wing shape than the age-related shift in the center of mass. In such cases, one may not be able to observe sex- and age-dependent changes in the wing tip shape. Thus, one can predict that similar changes in the wing tip shape would be more frequently observed in birds with backward-shifted wingtips. This hypothesis should be more rigorously tested with more extensive sampling of avian species. In addition, it should be examined whether males experience breeding-related mass change and impaired flight performance due to mass increase, in order to better understand the magnitude of differential selective pressure between sexes due to breeding on wing shape variation.

Our results suggest that juvenile magpies, who are more likely to engage in flapping-dominated dispersal flights, may not benefit from the aerodynamic advantages of a longer P6 configuration observed under gliding conditions. Currently we do not have empirical data on the flight speed and distance of magpies during dispersal. However, given the relatively short natal dispersal distances—reported to average only 1.2 territories in European species^21^—we hypothesize that selective pressures for flight efficiency during dispersal are likely to be weak. Nonetheless, the apparent inefficiency of the forward-shifted wingtip in terms of L/D under our test conditions may not necessarily translate to reduced performance during flapping flight, where other parameters, such as wingbeat kinematics in combination with morphological traits, could play a more dominant role. This highlights the importance of expanding future studies to include both gliding and flapping flight performance to better understand the adaptive significance of wing morphology across life stages and behavioral contexts.

## Method

### Wing shape analysis

We collected the corpses of magpies hunted by regional Korean Electric Power Corporation (KEPCO) in the early March of 2008; every spring KEPCO conducts the annual extermination of magpies in those areas where magpies potentially cause short circuits by putting nest materials on electricity poles. Collection sites include five localities; Andong (36° 56’, 128° 7’), Cheorwon (38° 00’, 127° 5’), Daegu (35° 76’, 128° 9’), Kunsan (37° 40’, 127° 5’) and Uljin (36° 90’, 129° 4’). Since body mass might be sensitive to how the samples are processed after the collection, body mass was measured as soon as possible with digital scale to the nearest 0.1 g. Samples were then stored in the freezer (− 20 °C). After two months, we thawed the samples at room temperature for 7–8 h, measured tarsus, tail, and culmen lengths with Vernier callipers to 0.1 mm accuracy, identified the sex of the individuals by checking the presence of testes or ovaries, and severed the wings from the body at the joints of humerus and coracoid. The age was determined by the amount of black tip on the longest primary feather (for more details, refer to^37^) and the presence of glossiness at the black tip. Because magpies have a partial post-juvenile molt where all the body plumage and coverts except primary feathers are replaced^21^, all second-year birds (SYs) had juvenal primaries with long black tips. Prebasic molts are complete, and all the birds after their second year (ASYs) had non-juvenal primaries with short and glossy black tips.

All the wing-related traits were measured and calculated with severed wing samples. As the indices for general flight performance, we calculated wing loading and wing aspect ratio. Wing loading (g/cm^2^) was calculated as body mass divided by wing area, and wing area was measured from the photos taken with fully stretched single wing. Wing aspect ratio was calculated as *wing span*^*2*^*/wing area*. Since many of our samples had humerus fracture on either wing because they were shot in the air and fallen to the ground, we used double the length of a single wing (the length between the end of humerus and the tip end of longest primary feather) as the proxy for wing span. Statistical comparisons revealed that wing loading did not show any significant difference among the classes, but aspect ratio differed between sexes (F_1,145_ = 22.59, *P* < 0.0001) without any significant interaction between sex and age (Supplementary Fig. 11).

As for the indices of wing shape, we extracted three principal components (PCs) from primary distances (i.e. distances from wingtip to the tip of each primary feathers) of 9th to 2nd primary feathers (numbering starts from the most proximal primary). Magpies have a vestigial tenth primary (25% of the width and 37% of the length of the longest primary) and we did not include this feather in the analysis. In the dataset, we used primary distances standardized by the allometry with wing length (P*)^38–40^. P* was calculated from the following equation:

$${\text{P}}_{{{\text{ji}}}}^{*} = {\text{P}}_{{{\text{ji}}}} \left( {\frac{{{\text{l}}_{0} }}{{{\text{l}}_{{\text{i}}} }}} \right)^{{{\text{b}}_{{\text{j}}} }} .$$

In this equation, ${\text{P}}_{\text{ji}}$ is the original distance of primary *j* in the individual *i*, ${\text{l}}_{\text{i}}$ is the wing length of the individual *i*, ${\text{l}}_{0}$ is a standard wing length, and ${\text{b}}_{\text{j}}$ is the allometry coefficient of the primary *j* calculated from the growth model ${\text{P}}_{\text{ji}}={\text{a}}_{\text{j }}{\text{l}}_{\text{i}}^{{\text{b}}_{\text{j}}}$ where a_j_ is a parameter. Since the b values for the first primary distance did not converge, primary distances from second to ninth primaries were included in the dataset. For the standard wing length (l_0_), we used locality-specific least-square mean values of wing length. We did not use the wingtip shape indices that were designed for interspecific comparisons (e.g. Lockwood et al.^4^ indices), because, although these indices were also applied in intraspecific comparisons (e.g. Arizaga et al. 2006^41^), the difference in wing shape of individuals that belong to the same species can be too minute to be properly described by those indices. Thus, we used PCA-based methods for species-specific characterization of wing shape. In order to reduce measurement error, all the morphological variables were measured by one of the authors (J. Hwang) and primary distances were measured by one assistant (J. Oh). Details of the principal component analyses are provided in Supplementary Table 1 and associated figures.

We used general linear models in order to examine the effect of age and sex on the morphological parameters and wing shape indices. Since we were interested in identifying the age-related wing shape change in females and males, we conducted planned comparisons between SY and ASY birds in females and in males separately. All the morphological variables and PCs showed normal distributions (Kolmogorov–Smirnov tests; all had *P* values above 0.09). The sampling locality was included as a random variable. All the statistical analyses were performed with SAS ver 9.2 (SAS Institute, Cary).

### Force measurements

Force measurements of the wings were performed in an open-circuit wind tunnel (see Supplementary Fig. 2(a)). The open-circuit wind tunnel used in this study has a test section of 1 m (height) × 1 m (width) × 5 m (length). The turbulent intensity of the freestream in the wind tunnel is within 0.4%. The freestreams (*U*_∞_) in the wind tunnel were set to 3, 5, 7, 9, and 11 m/s within the actual magpie’s flight speed range. The corresponding Reynolds numbers (*Re* = *U*_∞_*c*/*ν*; *ν* = kinematic viscosity of air) based on the mean chord length (*c* = *A*/*s*; *A* = projected area, *s* = maximum span length) are approximately 23,000, 39,000, 54,000, 70,000, and 86,000. Two load cells (CAS BCL-5L) were installed just below the wing to measure the lift (*L*_*W*_) and drag (*D*_*W*_) of the wings. The calibration curve of the loadcell is linear within the maximum capacity of 50 N. The acquisition rate of the force data was 1000 Hz and was measured for 120 s to obtain fully converged average data. The force measurement of each case was repeated two times and the average value was used. Uncertainties of lift and drag forces are within 1.3%. The force data measured in the load cell was transmitted to the PC through an A/D converter and an amplifier. An end plate was installed between the wind tunnel wall and the wing to eliminate the boundary layer effect of the wind tunnel wall. A rotational stage was installed just below the wing model to control the angle of attack of the wing. The angle of attack is defined as the angle between the freestream direction and the line connecting the leading edge and the trailing edge from the view of the wing root without wind conditions. The range of angle of attack (*α*) in which the aerodynamic performance was measured was from − 30° to 50° with an interval of 4°. The blockage ratio of the wing reached a maximum of 2.5% at *α* = 50°.

The number of evaluated magpie wings is three and is named wing 1, wing 2, and wing 3, respectively. Here, wing 1 is the left wing of an adult female specimen, while wings 2 and 3 are the left wings of adult male specimens. The wing models were prepared by cutting the shoulder joint between the humerus and the scapula and dried in a gliding posture. For each wing models, feather lengths from P5 to P8 were adjusted by a feather length adjustment device (FLAD). Details of the FLAD are described in Supplementary Figs. 2(b) and 2(c). It was confirmed that the wings with the FLAD had almost the same aerodynamic performance as the natural wings over the range of angles of attack we measured (see Supplementary Fig. 10).

Drag measurements of the magpie body were performed in the wind tunnel where the forces of the wing were measured. The magpie body was positioned such that both the body axis and tail were aligned with the freestream direction. The tail was fixed at a spread angle of approximately 30°, which falls within the range of 10° to 50° typically observed in flying magpies at speeds of 4–10 m/s^24^. Here, details of experimental setup and results for a body drag measurement are described in Supplementary Fig. 8. To measure the body drag of the magpie, the magpie body of wing 1 was dried without wings after both shoulder joints. The body drag coefficient (*C*_*D,b*_ = *D*_*b*_/0.5*ρU*_∞_^2^*A*_*b*_,*A*_*b*_ = frontal area of the magpie body) was measured from 3 to 11 m/s with an interval of 2 m/s. The body model was fastened by a stainless-steel shaft to maintain its posture so that the line from the eye to the tip of the tail was parallel to the horizon.

### Flight performance estimation

The flight performances of magpies are estimated for various wingtip shapes in the helical-descending flight condition. It should be noted that the flight trajectory analysis was performed following the method utilized in Lentink et al.^23^. The force applied to the magpie consists of weight, total drag, and total lift. These forces were evaluated separately from the body and wings and defined as follows:

- Drag: $D = D_{{\text{b}}} + D_{{\text{w}}}$, where* D*_b_ is body drag;
- Lift: $$L = 2L_{{\text{w}}}$$;
- Weight: $W = W_{{\text{b}}} + 2W_{{\text{w}}}$, where *W*_b_ is the body weight.

In the range of free-stream velocities from 3 to 11 m/s, the body drag coefficient (*C*_*D,b*_) remains nearly constant, showing minimal dependence on the free-stream velocity, as illustrated in Supplementary Fig. 8(b). Consequently, *C*_*D,b*_ was approximated as the average value of 0.395 for evaluating the flight performances. Utilizing the lift, drag, and weight, the flight parameters, including glide angle (*γ*), bank angle (*μ*), and turning radius (*R*), were calculated for various glide speeds (*V*) and angles of attack. The calculation of each flight parameter was conducted using the following equations:

- Glide angle: $$\gamma = {\text{arcsin}}\left( \frac{D}{W} \right)$$;
- Bank angle: $$\mu = {\text{arccos}}\left( {\frac{{\sqrt {W^{2} - D^{2} } }}{L}} \right)$$;
- Turning radius: $$R = \frac{{mV^{2} }}{{W^{2} }}\left( {\frac{{W^{2} - D^{2} }}{{\sqrt {L^{2} + D^{2} - W^{2} } }}} \right)$$.

Flight trajectories were analyzed limitedly within several conditions as follow: 1) *W* > *D*; 2) *L*^2^ + *D*^2^ > *W*^2^; 3) *R* > 0. It is calculated by dividing it into straight-flight and turning-flight indices calculated from the flight trajectory. Turning flight indices consist of turning angle (1/(*R*tan*γ*)), angular velocity (*V*cos*γ*/*R*), and curvature (1/*R*). Turning performances were calculated in not much long *R* (less than 10 m) and not much high *γ*. (less than 45$^\circ$). To analyze the indices of straight-flight performance, the turn radius was set to have an infinite value. Straight-flight indices are composed of glide ratio (1/tan*γ*), glide duration (1/(*V*sin*γ*)), and horizontal speed (*V*cos*γ*). To construct Fig. 4, the maximum values of indices at each glide speed were derived within the angle of attack range where the aerodynamic forces were measured (− 30$^\circ$ to 50$^\circ$).

## Supplementary Information

Supplementary Information 1.

Supplementary Information 2.

## Data Availability

The datasets used and/or analysed in this study are uploaded as a supplementary material.
