# Supplementary material for: Empirical evidence for the functional benefit of intra-specific wing shape variation in a sedentary bird, the Oriental Magpie (Pica serica)
Source: Sci Rep. 2025 Aug 12;15:29495. doi: 10.1038/s41598-025-13894-4 (PMC12343764; doi:10.1038/s41598-025-13894-4)
Supplement: Supplementary file 1 — Supplementary Information 1. [file 41598_2025_13894_MOESM1_ESM.docx]

**Supplementary material**

Supplementary Fig. 1 | Change in the aerodynamic center due to a distal shift of the wingtip

Supplementary Fig. 2 | Experimental setup for force measurements of wings and details of feather length adjustment device (FLAD)

Supplementary Fig. 3 | Changes in primary feather length according to case number

Supplementary Fig. 4 | Effects of wingtip shape on lift and drag coefficients for wing 1

Supplementary Fig. 5 | Effects of wingtip shape on lift and drag coefficients for wing 2

Supplementary Fig. 6 | Effects of wingtip shape on lift and drag coefficients for wing 3

Supplementary Fig. 7 | Variations in the maximum lift coefficient with the wingtip shape of wings 2 and 3

Supplementary Fig. 8 | Drag coefficient of the magpie body model

Supplementary Fig. 9 | Effects of wingtip shape on the angular velocity and curvature for wing 3

Supplementary Fig. 10 | Drag-lift polars of the wings with and without the FLAD

Supplementary Fig. 11 | Intraspecific variation in aspect ratio and wing loading in the Oriental Magpie

Supplementary Table 1 and associated figures | Result of the principal component analysis for wing shape characterization based on normalized primary distances

Supplementary Table 2 | Dataset used in this study (uploaded separately)

Supplementary Fig. 1(a) illustrates a simplified bird wing model used to evaluate the aerodynamic center. This simplified model comprises a rectangular section representing the proximal wing region, including the secondary feathers and coverts, and ten trapezoidal sections (with a taper ratio of 0.5 and *c*_Pt_ = 0.5*c*_P_) representing the primary feathers. The aerodynamic center of the entire wing model is calculated as the weighted average of the aerodynamic centers of individual sections (Diehl 1942, Vogeltanz 2016):


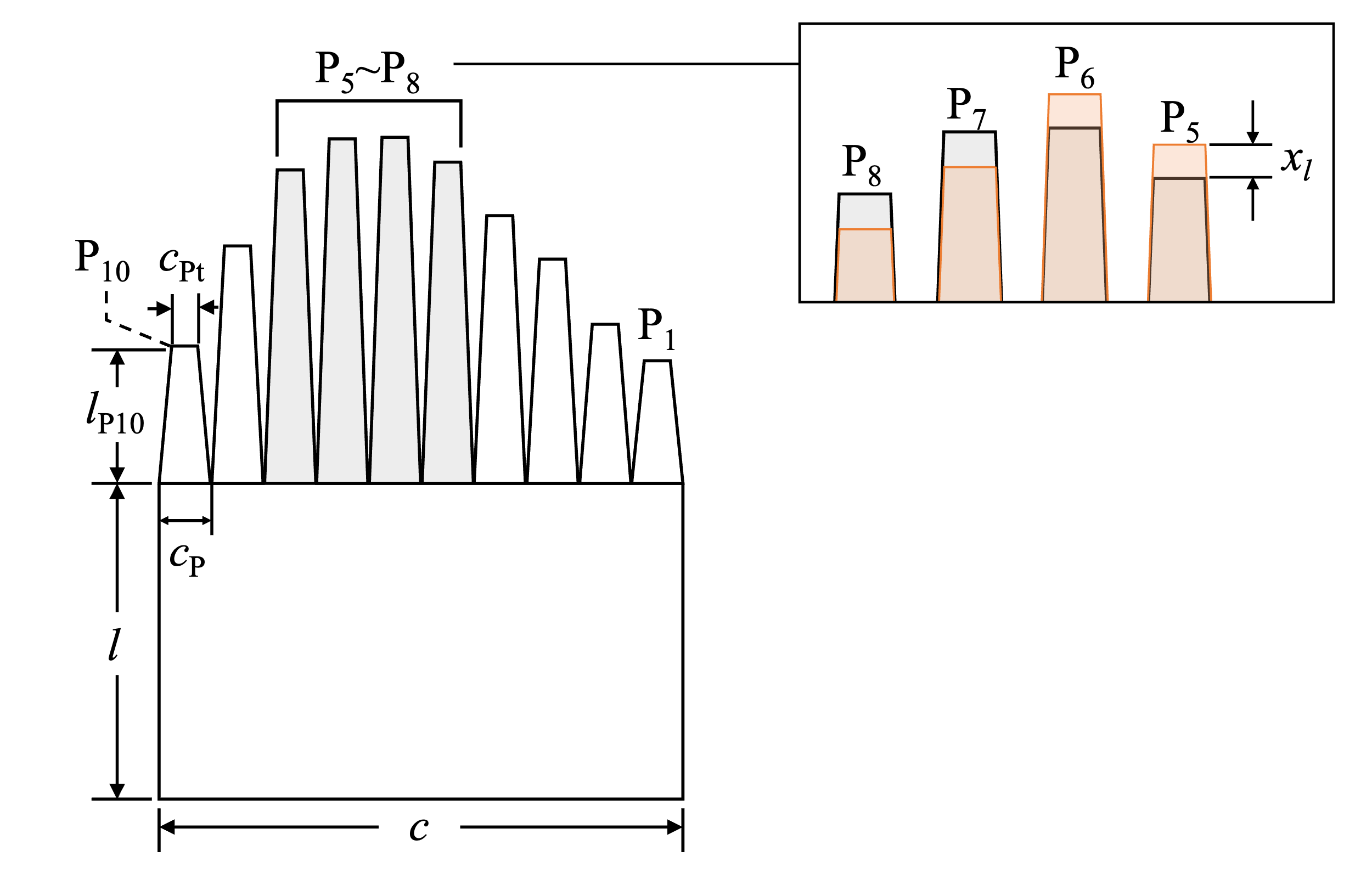


**Supplementary Fig. 1 |** **Change in the aerodynamic center due to a distal shift of the wingtip** Schematic of the simplified wing model. The distal shift of the wingtip is implemented by changing the lengths of the gray tapered wing sections (see inset).

|  | $\text{x}_{\text{ac,total}}\text{= }\frac{\sum_{\text{i}} \text{A}_{\text{i}}\text{∙}\text{x}_{\text{ac,}\text{i}}}{\sum_{\text{i}} \text{A}_{\text{i}}}$ | (1) |
| --- | --- | --- |
|  |  |  |

where *A_i_* is the area of the *i*^th^ wing section, and *x*_ac,_*_i_* is the aerodynamic center of the corresponding section.

To shift the wingtip shape distally, the lengths of feathers P5–P8, which strongly influence the tip morphology, are altered (in a manner consistent with modifications applied to actual wing models, as described later). The distal shift of the wingtip by a distance *x*_l_ is defined such that the anteriorly positioned feathers P7 and P8 are shortened by *x*_l_, while the posteriorly positioned feathers P5 and P6 are lengthened by *x*_l_.The corresponding changes in area for each feather are given by:

|  | ${\text{∆}\text{A}}_{\text{P}\text{n}}\text{ = 0.75}\text{c}_{\text{P}\text{n}}\text{∆}\text{l}_{\text{P}\text{n}}\text{,}\left\{ \begin{aligned} \text{∆}\text{l}_{\text{P}\text{n}}\text{ }\text{=}{\text{ }\text{x}}_{\text{l}}\text{ for }\text{n}\text{=5 and 6} \\ \text{∆}\text{l}_{\text{P}\text{n}}\text{ }\text{=}\text{ -}\text{x}_{\text{l}}\text{ for }\text{n}\text{=7 amd 8} \end{aligned} \right.$ | (2) |
| --- | --- | --- |
|  |  |  |

where *n* is the primary feather number (5–8).

The aerodynamic center in the *x*-direction for each tapered section and the rectangular section is assumed to lie at one-quarter of the chord length from the leading edge of each wing section. Therefore, the *x*-coordinate of the aerodynamic center for each tapered section is defined as:

|  | $\text{x}_{\text{ac,P}\text{n}}\text{ = (10-}\text{n}\text{)}\text{c}_{\text{P}\text{n}}\text{+1/4}\text{ }\text{c}_{\text{P}\text{n}}$ | (3) |
| --- | --- | --- |
|  |  |  |

where n is the primary feather number (1–10).

Accordingly, the change in the aerodynamic center of the entire wing resulting from a distal shift of the wingtip by *x*_l_ can be calculated as:

|  | $\text{∆}\text{x}_{\text{ac,total}}\text{ = }{\text{(∆}\text{A}}_{\text{P5}}\text{x}_{\text{ac,P5}}\text{+}{\text{∆}\text{A}}_{\text{P6}}\text{x}_{\text{ac,P6}}\text{+}{\text{∆}\text{A}}_{\text{P7}}\text{x}_{\text{ac,P7}}\text{+}{\text{∆}\text{A}}_{\text{P8}}\text{x}_{\text{ac,P8}}\text{)/}\text{A}_{\text{total}}\text{ = 3}\text{x}_{\text{l}}{\text{c}_{\text{P}}}^{\text{2}}\text{/}\text{A}_{\text{total}}\text{ }$ | (4) |
| --- | --- | --- |
|  |  |  |

This result demonstrates that as the wingtip shape is shifted distally, the aerodynamic center of the entire wing progressively moves backward.


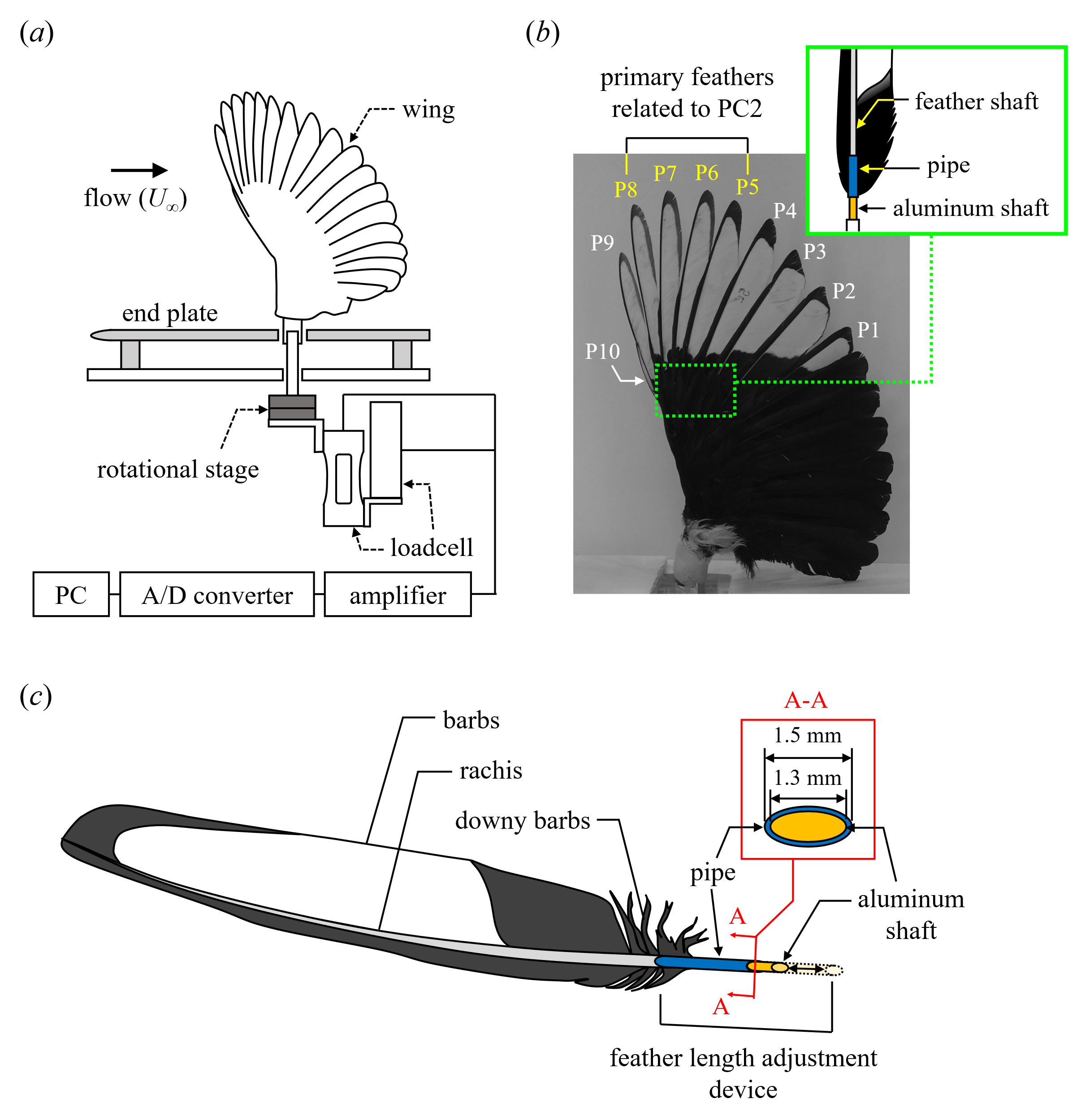


**Supplementary Fig. 2 |** **Experimental setup for force measurements and feather length adjustment device (FLAD) system** (a) Schematic diagram of the experimental setup to measure the aerodynamic performance of the wing model. (b) A photograph for detailed descriptions of the dried wing model. Here, the green-dashed square indicates the region where FLADs were installed and the inset of Supplementary Fig. 2(b) shows the schematic diagram of a feather with FLAD; (c) Details of a feather with FLAD.

Supplementary Fig. 2(a) shows the schematic diagram of experimental setup for measuring lift and drag coefficients of the wing model. Forces of the wing model were measured in a wind tunnel (Long Win Science & Technology, LW-3660), which has a test section with dimensions of 1 m (width) × 1 m (height) × 5 m (length). The maximum operational speed of the wind tunnel is 20 m/s, and the turbulent intensity is within 0.4%. The wing model is installed on the lower wall, which is 1.9 m away from the inlet of the test section (Supplementary Fig. 2(b)).

To accurately measure lift and drag of the wing model, two load cells were installed directly under the wing model. The load cells have a maximum capacity of 50N and have been linearly calibrated. The force data was acquired with the sampling rate of 1000 Hz and averged for 100 s. An end plate has been installed on the lower side of the test section where the wing model is installed. The end plate is a rectangle with a length of 0.9 m, a width of 0.9 m, and a height of 0.1 m. To prevent flow separation at the leading edge of the end plate, the leading edge section is designed to be streamlined. To adjust the angle of attack of the wing, a manual rotation stage has been installed directly beneath the wing. The force data was trasferred to PC throught amplifier and A/D converter.


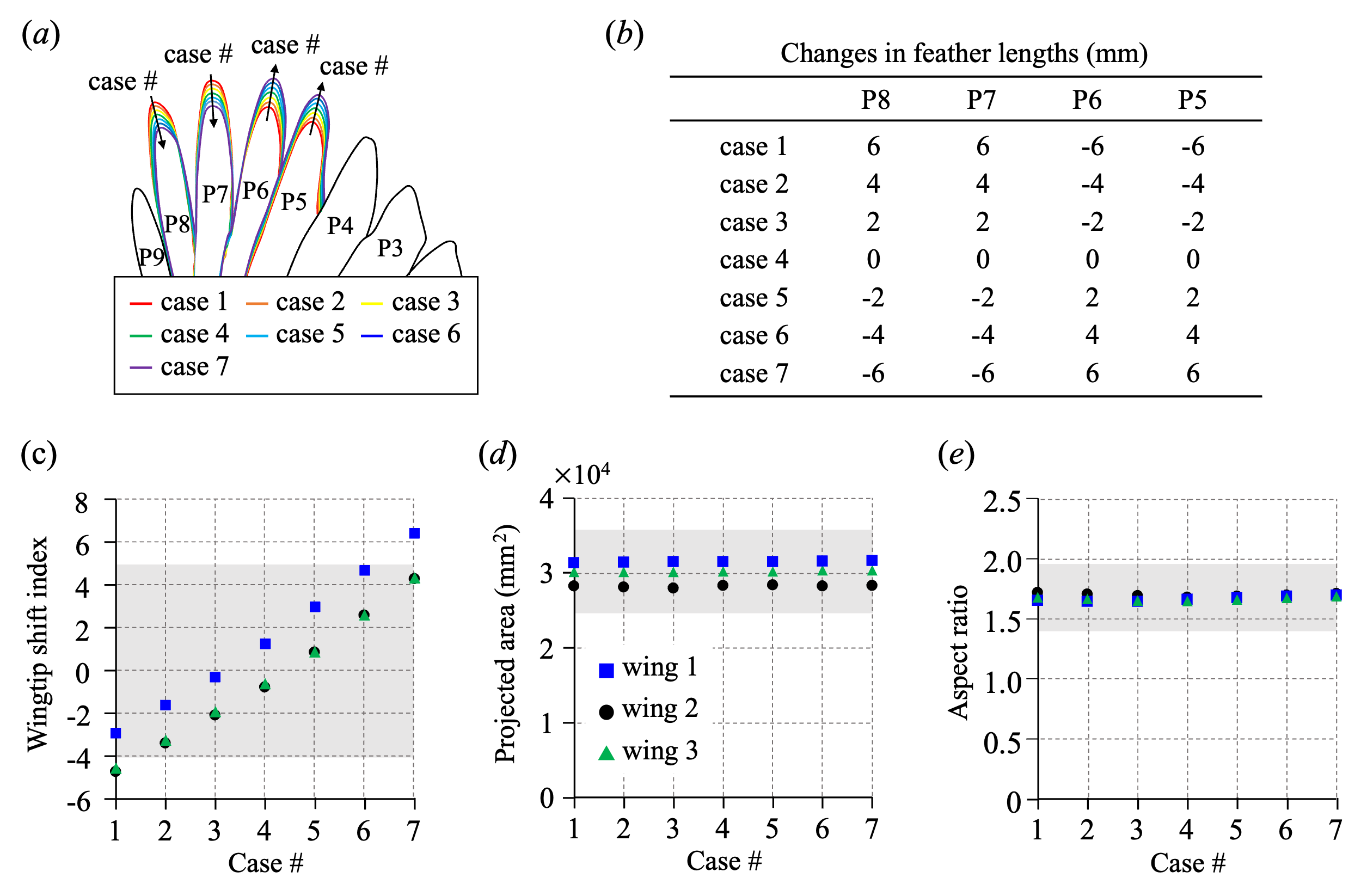


**Supplementary Fig. 3 | Changes in feather length for the wing model** (a) Variations in the wingtip shape depending on the case number. (b) Difference in feather lengths (from P5 to P8) compared to the natural feather lengths depending on the case number. (c-e) Variations in (c) wingtip shift index, (d) projected area, and (e) aspect ratio with the case number. Here, the gray regions show the parameter ranges of actual magpies.

The feather lengths within the wing models were adjusted by a feather length adjustment device (FLAD). The adjustments of feather length were conducted within the range of P5~P8, which are the feathers dominantly related to the wingtip shape. The calamus near the follicle of each feather was cut and the 18 mm calamus area of the feather shaft was replaced by the FLAD (see Supplementary Fig. 2(c)). FLAD consists of an aluminum shaft and a pipe, which was a part of the small antenna for smartphones. The cross-section shape of FLAD is elliptical so that it prevents any change in the natural angle of primary feather’s attachment to the main body of the wing. The length of the FLAD is adjusted by the aluminum shaft entering the pipe. The aluminum shaft and pipe have an elliptical cross-section with maximum diameters of 1.6 mm and 1.3 mm, respectively. The FLAD was fastened through a thin tape after the feather length was adjusted. To minimize undesired effects of the device on aerodynamic forces compared to conventional wings, the devices were installed while maintaining the vane shape of the feather and placed nearby the feather root to prevent exposure beyond the primary cover (see the inset of Supplementary Fig. 2(b)).

The wing shapes were divided into seven cases by adjusting the lengths of primary feathers (from P5 to P8). Supplementary Fig. 3(a) shows the variations in the wingtip shape for wing 1 according to a case number. The length of the primary feather with FLAD can be adjusted within the range of ±6 mm as shown in Supplementary Fig. 3(b). It should be noted that case 4 refers to a natural wing shape. The lengths of the wingtip features (P7, P8) located near the leading-edge and wingtip features (P5, P6) near the trailing edge were adjusted in the opposite direction because they had opposite dominant effects on the PC2 value. As the case number increases, P8 and P7 gradually shorten, while P6 and P5 gradually lengthen, resulting in a backward-shifted wingtip shape. Therefore, in all wings, the wingtip shift index increases linearly as the case number increases (Supplementary Fig. 3(c)). Depending on the case number, the wingtip shift index of wing 1 ranges from approximately -3 to 7, while wings 2 and 3 have a range of approximately -5 to 5. Note that these ranges were set to resemble the wingtip shift index ranges of actual magpie wings (see gray region in Supplementary Fig. 3(c)). On the other hands, the projected area and aspect ratio remain nearly constant across all wings, regardless of changes in the case number.


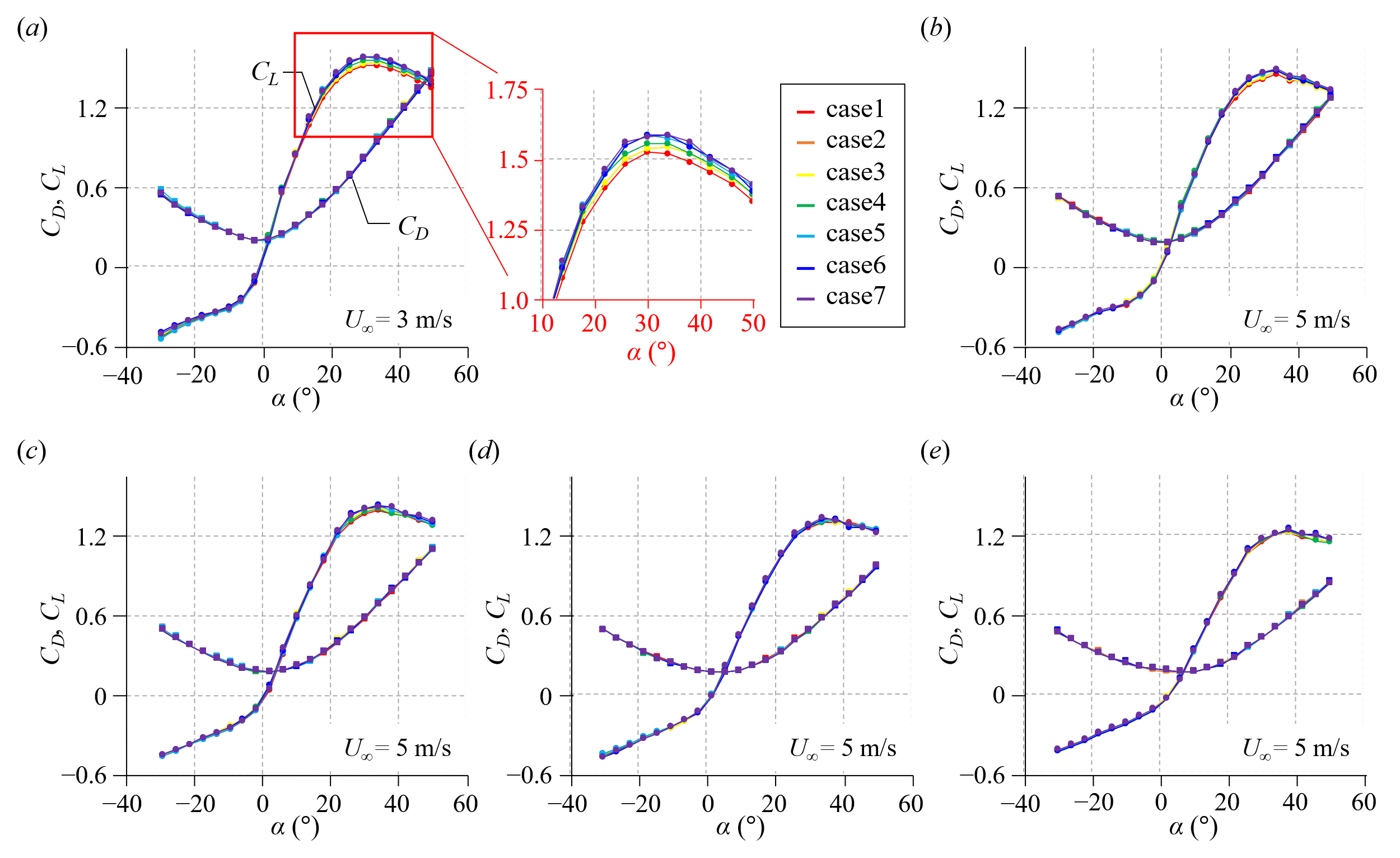


**Supplementary Fig. 5 | Effects of wingtip shape on lift and drag coefficients for wing 2** Change in force coefficients depending on angles of attack in all the case numbers of wing 2: *U_∞_* = (a) 3 m/s; (b) 5 m/s; (c) 7 m/s; (d) 9 m/s; (e) 11 m/s.


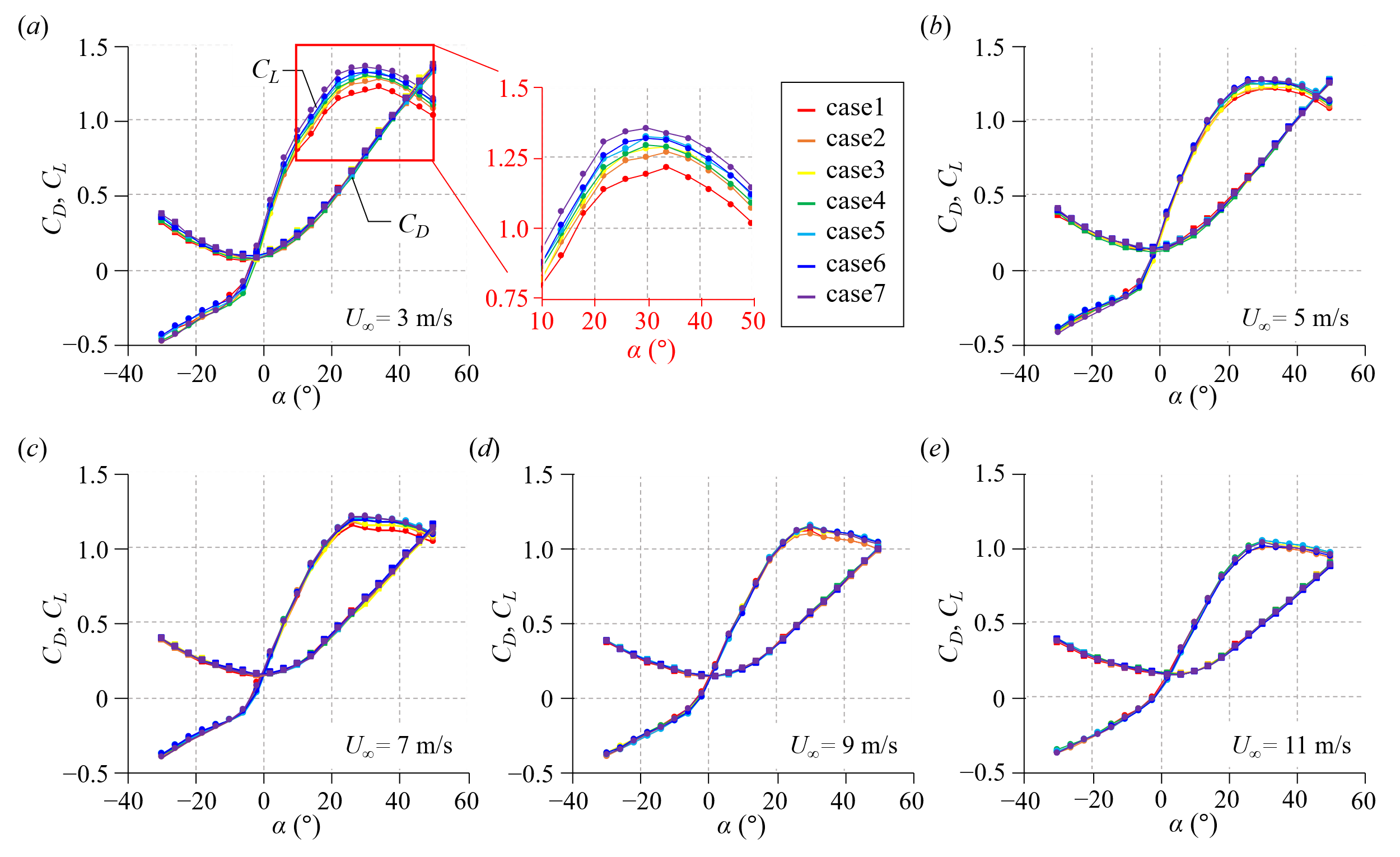


**Supplementary Fig. 4 | Effects of wingtip shape on lift and drag coefficients for wing 1** Change in force coefficients depending on angles of attack in all the case numbers of wing 1: *U_∞_* = (a) 3 m/s; (b) 5 m/s; (c) 7 m/s; (d) 9 m/s; (e) 11 m/s.


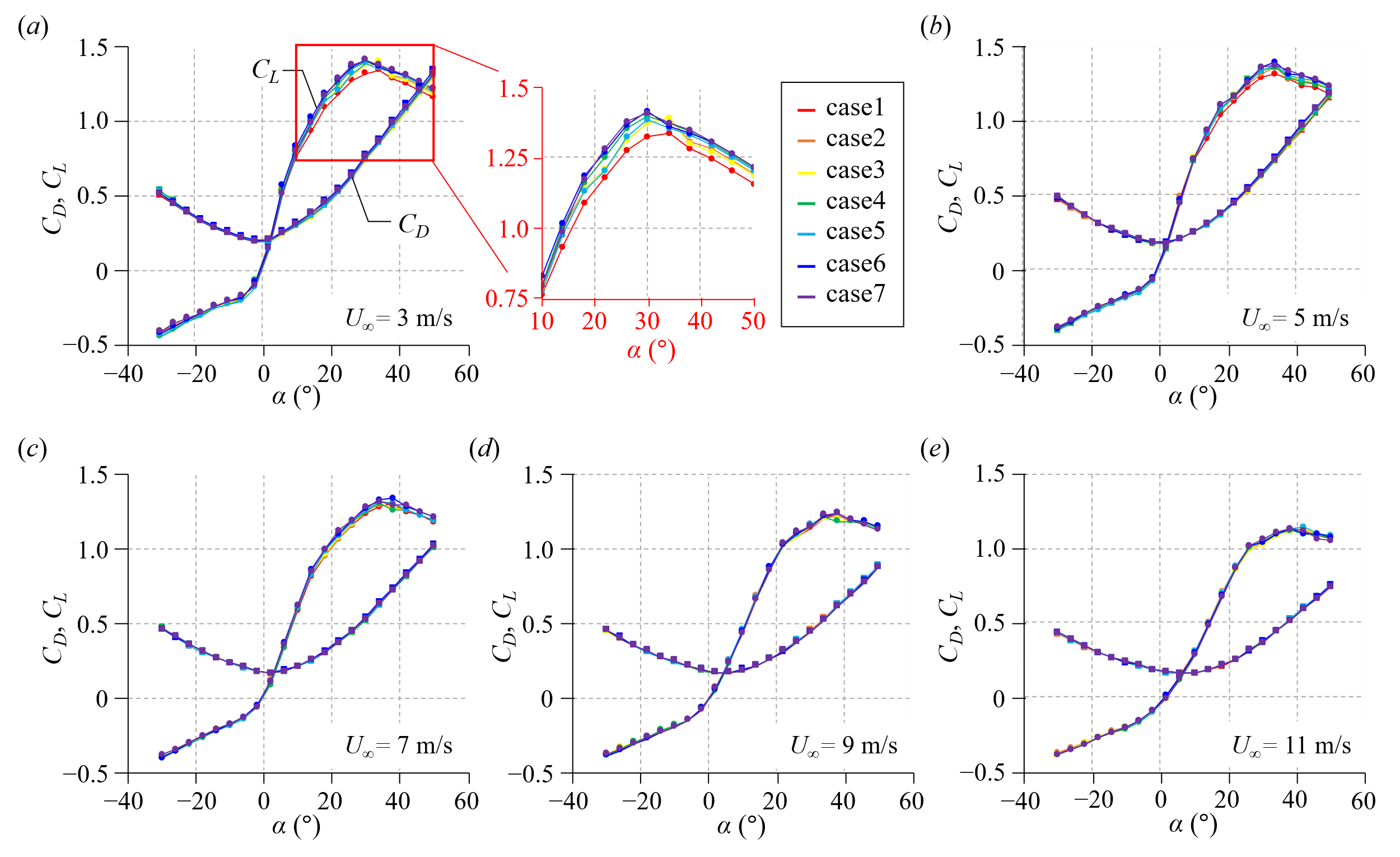


**Supplementary Fig. 6 | Effects of wingtip shape on lift and drag coefficients for wing 3** Change in force coefficients depending on angles of attack in all the case numbers of wing 3: *U_∞_* = (a) 3 m/s; (b) 5 m/s; (c) 7 m/s; (d) 9 m/s; (e) 11 m/s.

Supplementary Figs. 4, 5, and 6 show the variation of drag and lift coefficients with respect to the angle of attack for wings 1, 2, and 3. Supplementary Figs. 7 (a) and 7(b) show the variations in the maximum lift coefficient with the wingtip shift index at various *U_∞_*s for wings 2 and 3. Supplementary Fig. 7(c) shows the variation of slope of increase in the maximum lift coefficient (*s*), defined as the slope of the linear fitting line of *C_L,max_* along the PC2 value, according to *U_∞_*. At *U_∞_* = 3 m/s, for all wings, the lift coefficient gradually increases with increasing case number (i.e., increasing wingtip shift index) within a high angle of attack range. The maximum lift coefficient of wings 1, 2, and 3 increases by up to approximately 12%, 4%, and 5%, respectively, as the case number increased from case 1 to case 7. As the free-stream velocity increases, however, it is observed that the wingtip shape does not significantly affect drag and lift coefficients for all wings. For instance, at a high *U_∞_* of 11 m/s, wings 1, 2 and 3 with the highest wingtip shift index (i.e., case 7) show only a slight increase in lift coefficient at 30$^{\circ}$ to 3%, 1.8% and 2.9%, respectively, even when compared to the wings with the lowest wingtip shift index (i.e., case 1). For wings 2 and 3, therefore, there is a tendency for the value of *s* to decrease with increasing *U_∞_* (see Supplementary Fig. 7(c)). It should be noted that this trend is similar to the variation observed in the variation in the *s* value of wing 1 explained in Fig. 2(f). On the other hands, there is no significant difference in drag regardless of the change in the case number.

Supplementary Fig. 8(a) illustrates a schematic diagram of the experimental setup for measuring body drag. The body model, separated from the shoulder joint, was mounted, affixing it to the aluminum shaft near the tailbone. It should be noted that the body part of the specimen corresponding to wing 1 was utilized to measure drag force. The aerodynamic drag of the body model was measured in the same wind tunnel used to measure the aerodynamic performance of the wings. The body model is centrally positioned in the cross-sectional view of the test section, located 2.4 m away from the inlet of the test section. The body model was fixed to ensure that this longitudinal axis of body, the line connecting the eye to the tail tip, was parallel to the direction of the free-stream flow. The load cell was installed directly behind the body model to directly measure aerodynamic drag. The force data is transmitted to the computer through an amplifier and A/D converter. The force data was measured with a sampling rate of 1000 Hz and converged for 100 s. The body drag was measured at intervals of 2 m/s glide speed, ranging from 3 m/s to 11 m/s. Supplementary Fig. 8(b) shows the variations in the drag coefficient with glide speed of the body model. As the velocity increases from 3 m/s to 11 m/s, the drag coefficient decreases slightly, but it remains at a nearly consistent level. The average drag coefficient of the body model from 3 m/s to 11 m/s is 0.395. The body drag coefficient results in this study are roughly similar to the trend of the drag coefficient of a body model for a swift as a function of *U_∞_*, observed by Lentink et al. (2008).


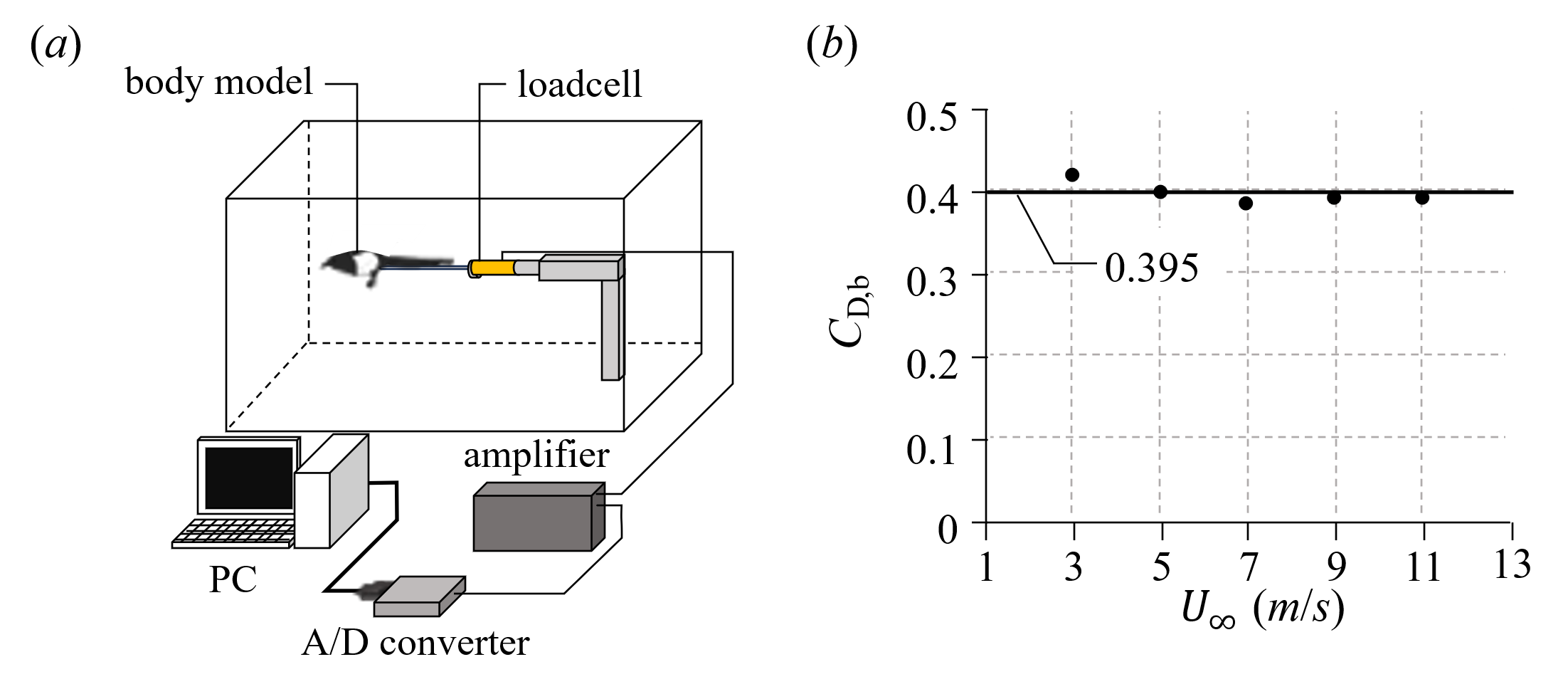


**Supplementary Fig. 8 | Drag coefficient of the magpie body model** (a) Schematic diagram of the experimental setup to measure the body drag. (b) Variation of the drag coefficient of the body model of wing 1 with the free-stream velocity. Here, the solid line means the drag coefficient of the body model averaged along the free-stream velocity.


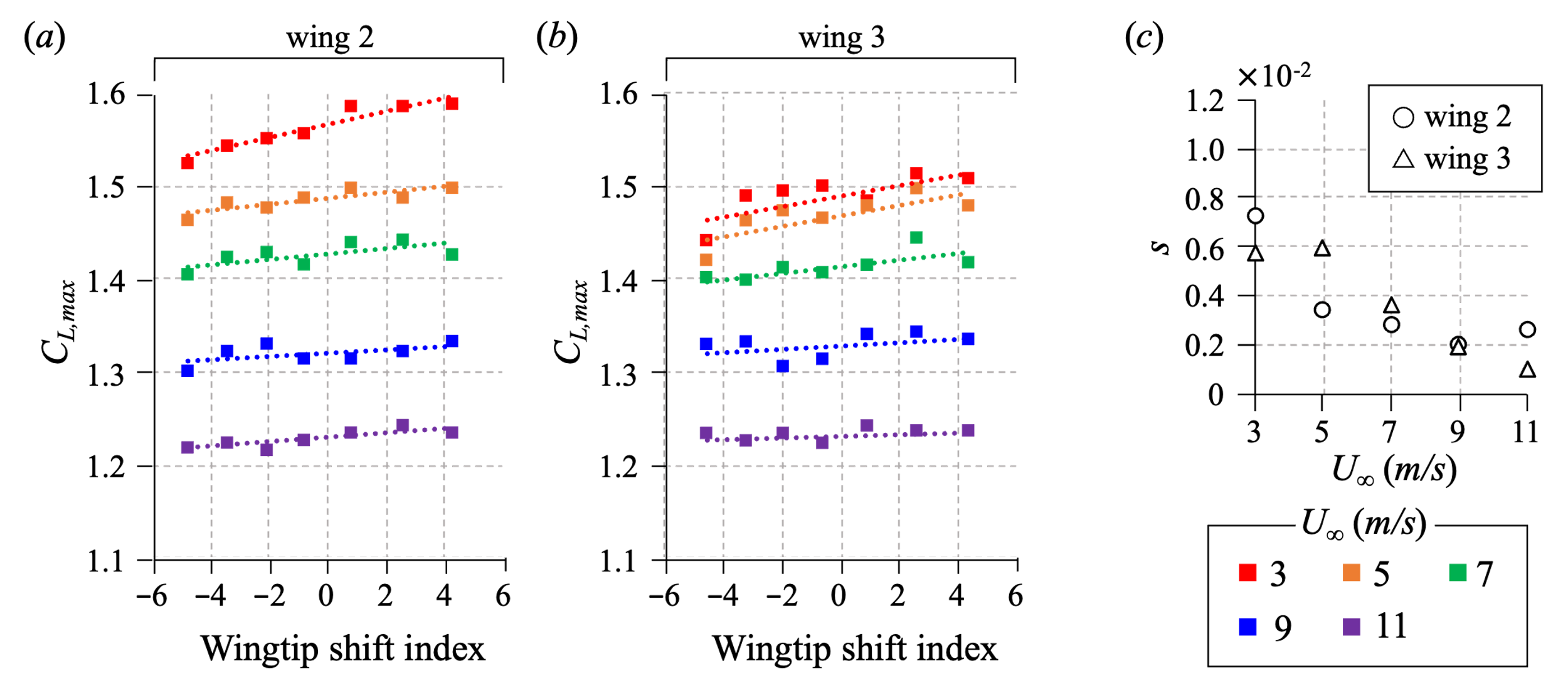


**Supplementary Fig. 7 | Variations in the maximum lift coefficient with the wingtip shape of wings 2 and 3** (a,b) Variations in the maximum lift coefficient with the wingtip shift index at various glide speeds for (a) wing 2 and (b) wing 3. (c) Variations in slope of increase in the maximum lift coefficient (*s*) with the glide speed for wings 2 and 3.

Supplementary Fig. 9 illustrates normalized angular velocity and normalized curvature contours for wing 1 across various case numbers. It should be noted that both angular velocity and curvature are dimensionless with respect to the natural feather length (i.e., case 4). Therefore, values lower than 1 indicate lower flight performance compared to the natural feather length, while values greater than 1 indicate higher flight performance. In the case of angular velocity, as the case number increases (i.e., as the wingtip shifts backward) in the low-glide-speed and high-angle-of-attack regions, higher angular velocity is observed. For example, at 6 m/s, case 7 exhibited a maximum about 30% higher angular velocity compared to case 1. However, it was challenging to observe a clear trend in angular velocity with respect to the wingtip shift index as the glide speed increased. Supplementary Fig. 9(b) depicts contour plots of normalized curvature at various case numbers. Higher curvatures are observed in the low-speed and high-angle-of-attack regions as the wingtip shifts backward. As the angle of attack increases, furthermore, there is no clear trend in curvature based on wingtip shape. It should be noted that the trends in angular velocity and curvature with respect to PC2 values align with the variation in turning angle shown in Fig. 3.

**Supplementary Fig. 9 | Effects of wingtip shape on the angular velocity and curvature for wing 1** Change in (a) normalized angular velocities and (b) normalized curvatures depending on glide speeds and angles of attack in turning-flight environment. Here, angular velocity* and curvature* are defined as the angular velocity and curvature normalized by those of the natural wingtip shape (i.e. case 4) under the same angle of attack and glide speed, respectively.


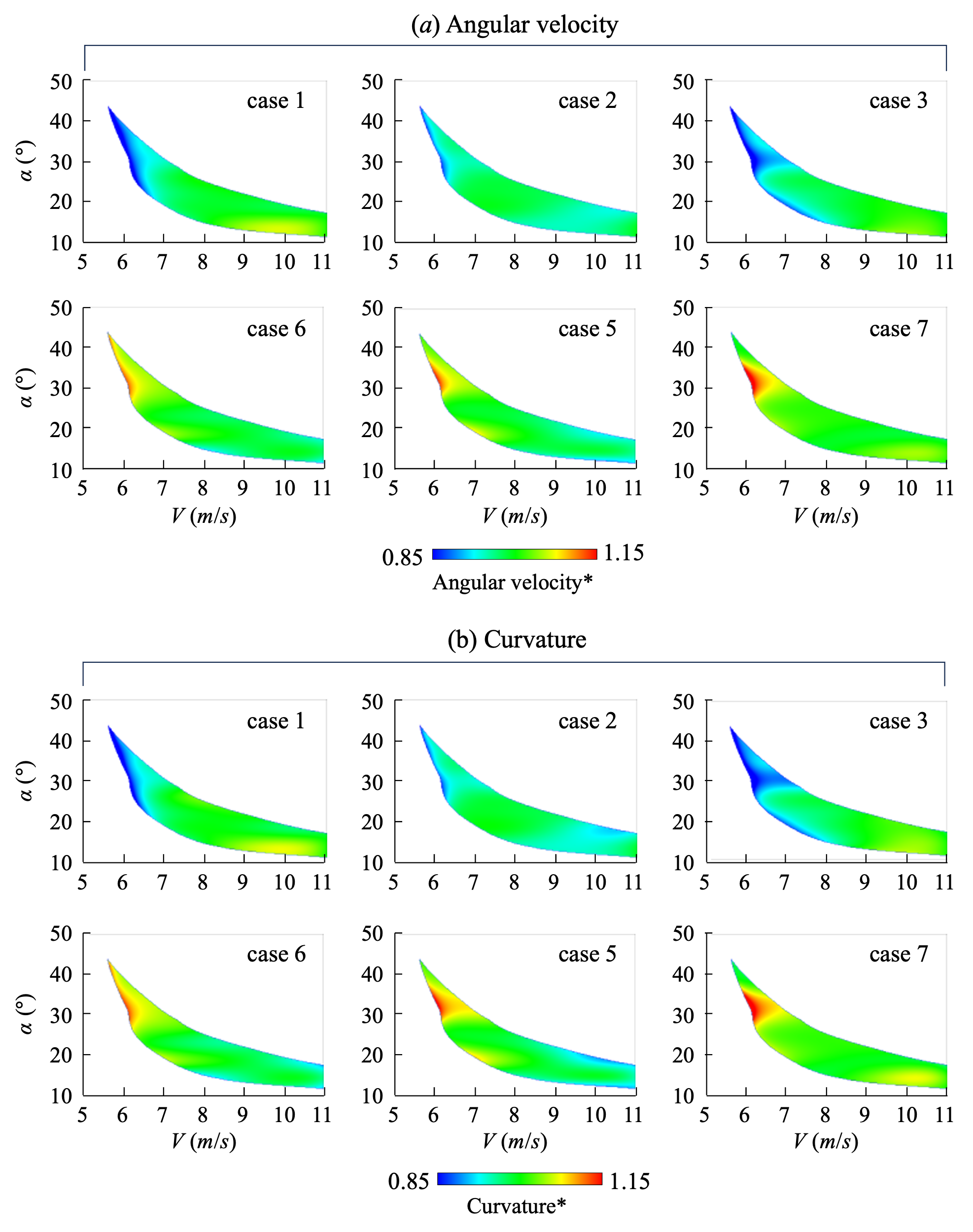


To investigate the impact of the FLAD, installed for adjusting the length of the wing feathers, on the aerodynamic performance of the wing, the aerodynamic performance before and after the installation of the FLAD was compared within the measured ranges of $\text{U}_{\text{∞}}$ and $\text{α}$, using the natural wingtip configuration (i.e., case 4) (Supplementary Fig. 10). It was observed that the aerodynamic performance remained similar between the conditions with and without the FLAD across all tested angles of attack and glide speed ranges. This indicates that the presence of the FLAD does not have a significant impact on the aerodynamic performance of the wing.


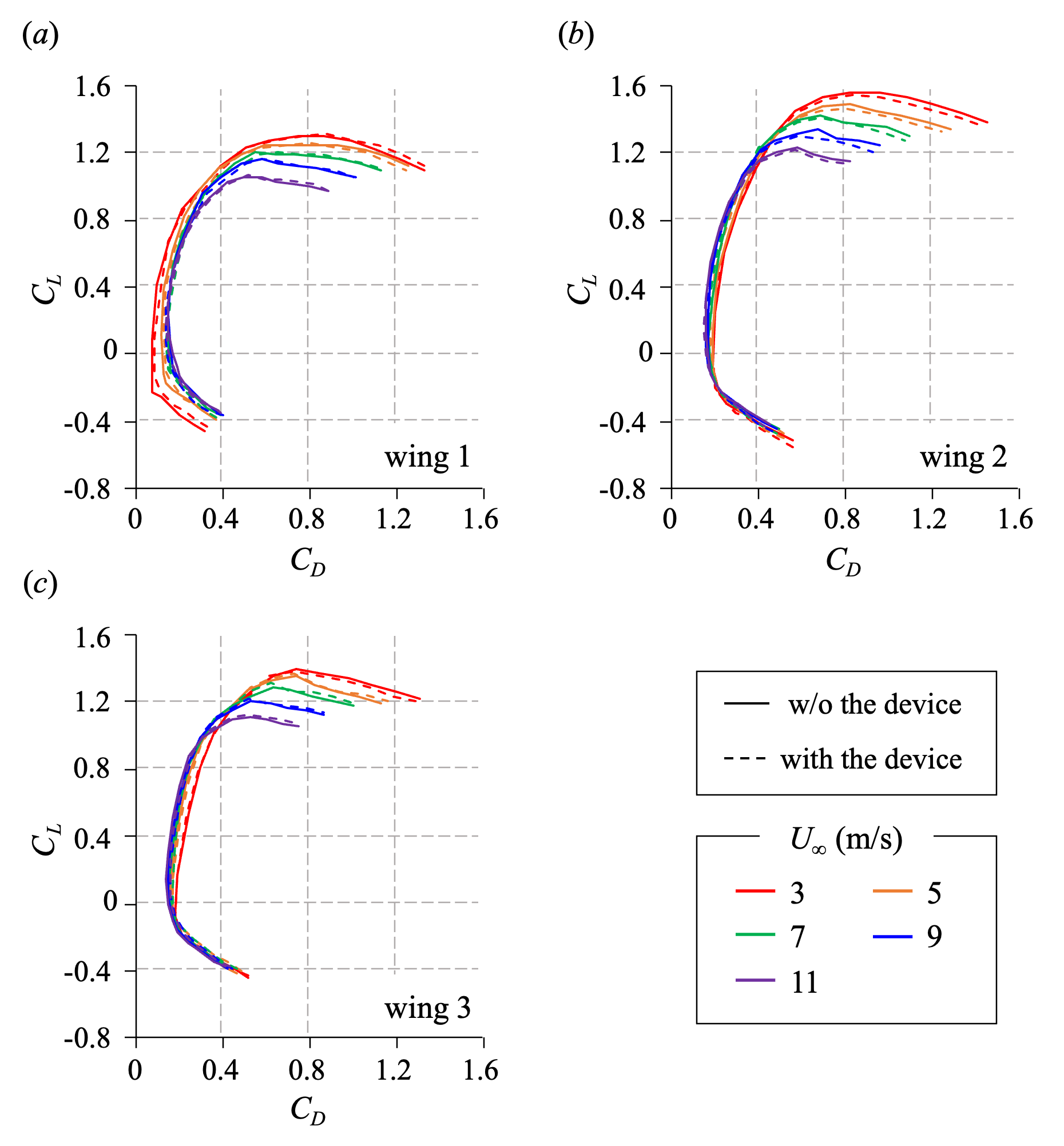


**Supplementary Fig. 10 | Drag-lift polars of the wings with and without the FLAD** Drag-lift polars at free-stream velocity from 3 m/s to 11 m/s for (a) wing 1, (b) wing 2, and (c) wing 3 with and without the FLAD, using the natural wingtip configuration (i.e., case 4).

**Supplementary Fig. 11 | Intraspecific variation in aspect ratio and wing loading in the Oriental Magpie.** Wing loading did not show any significant difference among the classes, but aspect ratio differed between sexes (F_1,145_=22.59, P<0.0001). In general males exhibited larger aspect ratio than the females; although the pattern shows age-related increase in aspect ratio in males, this was not significant (MS vs MA, adjusted P value =0.3). Significant post-hoc comparisons (with Bonferroni corrections) are marked with ‘*’, where ‘*’ denote 0.01<P<0.05, and ‘***’ denote P<0.001.


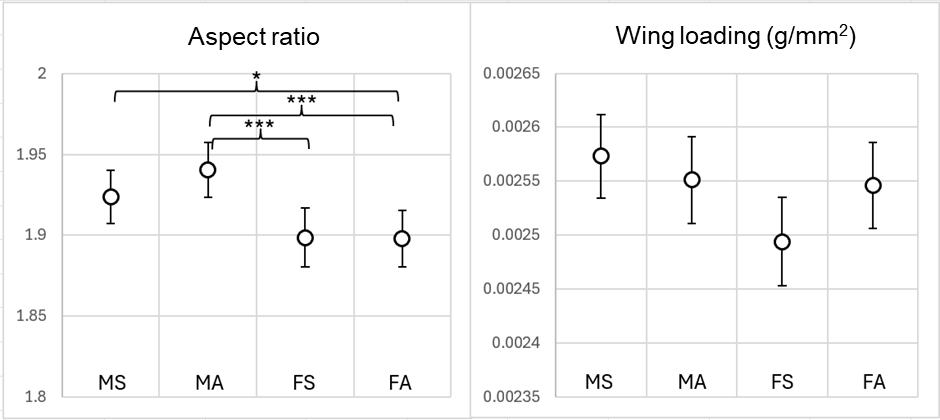


**Supplementary Table 1 and associated figures | Result of the principal component analysis for wing shape characterization based on normalized primary distances.** We used PC2, which is positively loaded with the primary distances in the distal part of the wing (closer to the leading edge) and negatively loaded with the distance of the 6^th^ primary. Thus, PC2 characterizes the shape of the distal part of the wing, with larger values indicating shorter outer primaries and longer P6 (i.e. smaller 6^th^ primary distance), and we used PC2 as a descriptor for wingtip shift. PC1, which consists of distances of inner primaries, exhibited significant sex difference with females having shorter inner primaries (larger distances for inner primaries) than males (F_1,107_=5.41, P=0.020). PC3, which is positively loaded with the distance of 9^th^ and 6^th^ primaries, exhibited significant age difference, with adults having larger values than juveniles (F_1,107_=8.61, P=0.004).

|  | **Eigenvectors** | | |
| --- | --- | --- | --- |
|  | **PC1** | **PC2** | **PC3** |
| **9^th^ primary distance*** | 0.174 | **0.475** | **0.452** |
| **8^th^ primary distance*** | 0.129 | **0.559** | 0.314 |
| **7^th^ primary distance*** | 0.144 | **0.494** | -0.253 |
| **6^th^ primary distance*** | 0.105 | **-0.421** | **0.667** |
| **5^th^ primary distance*** | **0.434** | -0.137 | 0.203 |
| **4^th^ primary distance*** | **0.524** | -0.097 | -0.011 |
| **3^rd^ primary distance*** | **0.513** | -0.054 | -0.156 |
| **2^nd^ primary distance*** | **0.442** | -0.097 | -0.350 |
| **Eigenvalue** | 3.024 | 1.987 | 1.035 |
| **Proportion** | 0.378 | 0.248 | 0.129 |
| **Cumulative** | 0.378 | 0.626 | 0.756 |


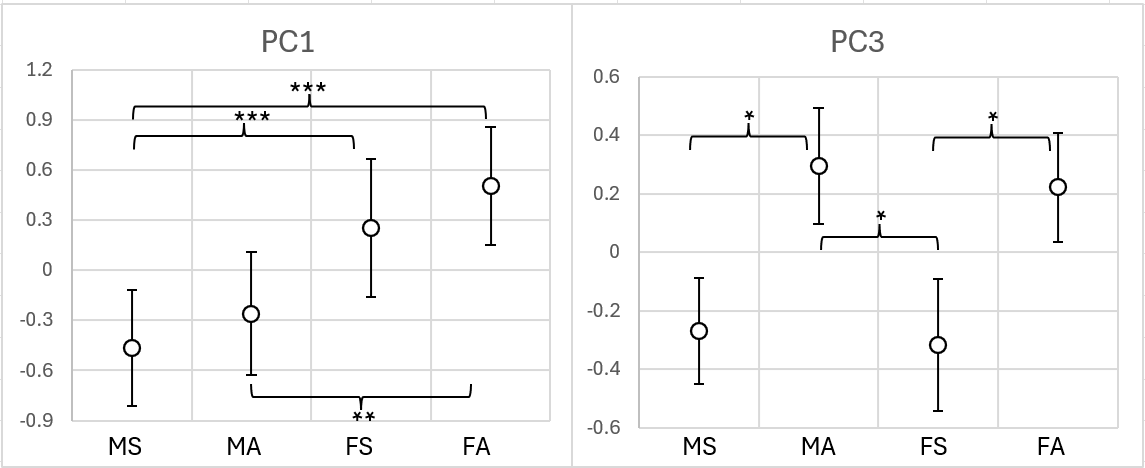


**References**

1. Lentink, D., Müller, U. K., Stamhuis, E. J., de Kat, R., van Gestel, W., Veldhuis, L. L. M., Henningsson, P., Hedenström, A., Videler, J. J. and van Leeuwen, J. L. How swifts control their glide performance with morphing wings. *Nature* **446**, 1082-1085 (2007).
2. Diehl, W. S. The mean aerodynamic chord and the aerodynamic center of a tapered wing. *NACA Tech. Rep.* **751** (1942).
3. Vogeltanz, T. Application for calculation of mean aerodynamic chord of arbitrary wing planform. *AIP Conf. Proc.* **1738**, 030053 (2016).
